# Viral pathogenesis of SARS-CoV-2 infection and male reproductive health

**DOI:** 10.1098/rsob.200347

**Published:** 2021-01-20

**Authors:** Shubhadeep Roychoudhury, Anandan Das, Niraj Kumar Jha, Kavindra Kumar Kesari, Shatabhisha Roychoudhury, Saurabh Kumar Jha, Raghavender Kosgi, Arun Paul Choudhury, Norbert Lukac, Nithar Ranjan Madhu, Dhruv Kumar, Petr Slama

**Affiliations:** 1 Department of Life Science and Bioinformatics, Assam University, Silchar, India; 2 Department of Biotechnology, School of Engineering and Technology (SET), Sharda University, Greater Noida, UP, India; 3 Department of Applied Physics, School of Science, Aalto University, Espoo, Finland; 4 Department of Microbiology, R. G. Kar Medical College and Hospital, Kolkata, India; 5 Health Centre, Assam University, Silchar, India; 6 Department of Urology and Andrology, AIG Hospitals, Gachibowli, Hyderabad, India; 7 Department of Obstetrics and Gynecology, Silchar Medical College and Hospital, Silchar, India; 8 Faculty of Biotechnology and Food Sciences, Slovak University of Agriculture in Nitra, Nitra, Slovak Republic; 9 Department of Zoology, Acharya Prafulla Chandra College, New Barrackpore, North 24 Parganas, West Bengal, India; 10 Amity Institute of Molecular Medicine and Stem Cell Research, Amity University, Noida, India; 11 Department of Animal Morphology, Physiology and Genetics, Faculty of AgriSciences, Mendel University in Brno, Brno, Czech Republic

**Keywords:** SARS-CoV-2 infection, COVID-19, ACE2, male reproduction, testicular damage, fertility

## Abstract

Coronavirus disease 2019 (COVID-19) has emerged as a new public health crisis, threatening almost all aspects of human life. Originating in bats, severe acute respiratory syndrome coronavirus 2 (SARS-CoV-2) is transmitted to humans through unknown intermediate hosts, where it is primarily known to cause pneumonia-like complications in the respiratory system. Organ-to-organ transmission has not been ruled out, thereby raising the possibility of the impact of SARS-CoV-2 infection on multiple organ systems. The male reproductive system has been hypothesized to be a potential target of SARS-CoV-2 infection, which is supported by some preliminary evidence. This may pose a global threat to male fertility potential, as men are more prone to SARS-CoV-2 infection than women, especially those of reproductive age. Preliminary reports have also indicated the possibility of sexual transmission of SARS-CoV-2. It may cause severe complications in infected couples. This review focuses on the pathophysiology of potential SARS-CoV-2 infection in the reproductive organs of males along with their invasion mechanisms. The risks of COVID-19 on male fertility as well as the differences in vulnerability to SARS-CoV-2 infection compared with females have also been highlighted.

## 1. Introduction

In early December 2019, several pneumonia cases of unknown aetiology were reported in Wuhan, China. Genome sequencing studies confirmed these to be the result of a novel viral infection named severe acute respiratory syndrome coronavirus 2 (SARS-CoV-2), causing coronavirus disease 2019 (COVID-19) [1]. As of 22 December 2020, the viral outbreak has spread globally across as many as 222 countries, thereby infecting more than 76 million people and causing over 1.6 million deaths [2]. SARS-CoV-2 mainly enters the cell by binding to angiotensin-converting enzyme 2 (ACE2), a receptor found predominantly on the surface of epithelial cells in the lungs [3]. This is believed to be the main reason behind the vulnerability of the respiratory system to SARS-CoV-2 infection. However, ACE2 is also expressed in various other tissues of the body, and as a result, there is a high probability of SARS-CoV-2 infection of other organ systems, including the digestive, urogenital, circulatory, central nervous and reproductive systems [4].

Due to the high expression of the ACE2 receptor in testicular tissue in both somatic and germ cells, such as seminiferous duct cells, Leydig cells, Sertoli cells and spermatogonia, there is increasing concern about the possible impact of SARS-CoV-2 infection on male fertility [5,6]. Moreover, ACE2-mediated SARS-CoV-2 invasion may lead to viral infection, which may also cause damage to testicular tissues [7]. This indicates that the testis is a potential target of SARS-CoV-2 invasion and that damage to testicular cells may severely hamper the process of spermatogenesis. A recent study reported significant impairment of sperm quality in a COVID-19 patient [8]. Moreover, young men, if infected, may be at a greater risk of testicular damage due to higher expression of the ACE2 receptor in comparison to patients more than 60 years of age, who show comparatively lower levels of expression and are hence less prone to such testicular damage [9]. Single-cell RNA sequencing data of adult human testes indicated a higher positive rate of ACE2 in infertile men. The authors further suggested that such men with reproductive disorders may be susceptible to SARS-CoV-2 infection through a pathway activated by ACE2 [9]. However, infection in testicular organs does not necessarily mean direct damage to sperm cells. In a recent study, a semen sample of only 15.8% of COVID-19 patients under surveillance was found to be positive for SARS-CoV-2 particles, even in recovering patients [10]. By contrast, *in situ* hybridization studies could not confirm the presence of any viral genetic material in testicular tissues, and the damage was attributed to the infiltration of inflammatory molecules in the testicular tissue during the immunological response of the virus [11]. Recently, another group of researchers have also reported the absence of SARS-CoV-2 in the semen and testis of men in the acute infection and recovery phases [12]. This review discusses the origin of SARS-CoV-2 and its mechanism of invasion along with potential infection of the reproductive system of the affected male.

## 2. SARS-CoV-2: history, origin and transmission

**Figure 1.**
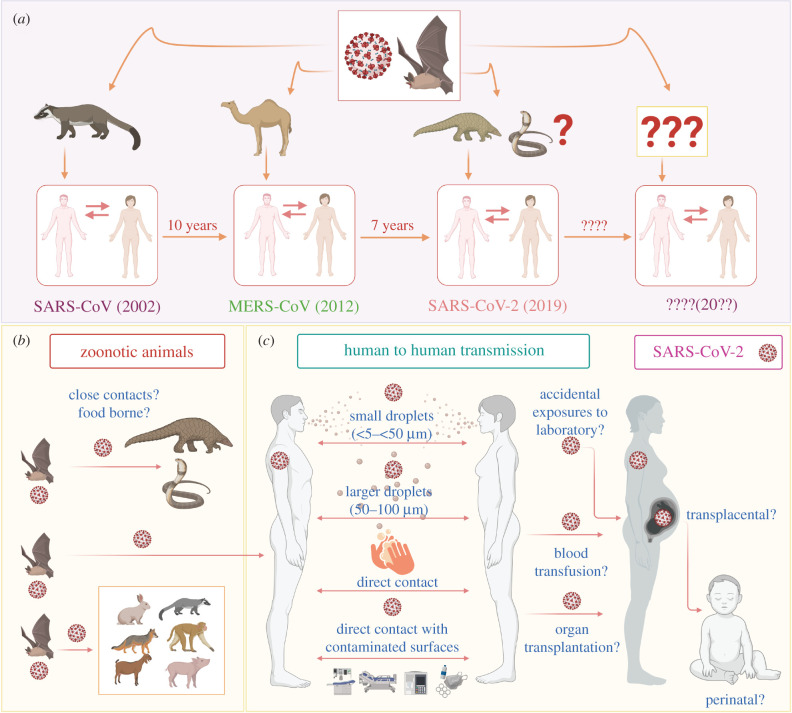
Origin of coronavirus and potential routes of transmission of SARS-CoV-2. (*a*) The origin of coronavirus. Similar to SARS and MERS, coronavirus is an emerging virus that has crossed the species barrier from wild animals to humans. The origin of SARS-CoV-2 is also suspected to be from an intermediate animal host, and the likelihood of crossing the species barrier for a fourth time cannot be ruled out. The current COVID-19 outbreak caused by SARS-CoV-2 has already been predicted and will also be contained sooner or later, similar to earlier outbreaks [15]. However, the real issue is how we plan to counter the next zoonotic CoV pandemic that is likely to occur in the next 5 to 10 years, if not sooner. (*b*,*c*) The potential routes of transmission of SARS-CoV-2. SARS-CoV-2 is alleged to have zoonotic (animal-to-human) origin with further human-to-human transmission [16], and the likelihood of food-borne transmission should be ruled out pending further investigation [17]. In addition, it can potentially be transmitted through direct contact, as in other respiratory viruses, such as by shaking contaminated hands or exposure to contaminated surfaces (fomite transmission). Nevertheless, other possible routes of SARS-CoV-2 transmission, such as accidental exposure to the laboratory, blood transfusion, organ transplantation [18], and transplacental and perinatal routes, need to be adduced more concretely. SARS-CoV: severe acute respiratory syndrome-related coronavirus, MERS-CoV: Middle East respiratory syndrome-related coronavirus, SARS-CoV-2: severe acute respiratory syndrome coronavirus 2.

Coronavirus was first observed during the mid-1930s [13], and the earliest human infection of coronavirus was documented in 1960 as a cold [14]. Much later, in 2002, a new species of coronavirus, originating from bats and transmitted to humans through palm civet cats as intermediate hosts, occurred and was named SARS-CoV (figure 1). In 2012, another coronavirus of bat origin, namely, Middle East respiratory syndrome coronavirus (MERS-CoV) emerged, with camel as an intermediate host [19]. Very recently, SARS-CoV-2 has caused the largest pandemic in recent human history and the first documented coronavirus pandemic of such a large magnitude [20].

Coronaviruses are divided into four genera, comprising α-, β-, γ- and δ-coronaviruses, of which only α- and β-coronaviruses are capable of infecting animals. SARS-CoV-2 is a β-coronavirus belonging to the family Coronaviridiae and the subfamily Orthocoronavirinae. It is an enveloped and non-segmented positive-sense, single-stranded RNA virus [21]. Human coronaviruses generally have zoonotic origins, and the genome sequence of SARS-CoV-2 shares 96.2% identity with the bat coronavirus RaTG13. This suggests that SARS-CoV-2 originated in bats and was transmitted to humans through unknown intermediate hosts (figure 1) in the Wuhan seafood market in China in December 2019 [22,23]. Metagenomic sequencing has revealed that pangolins might have acted as intermediate hosts between bats and humans because of the similarity of the pangolin coronavirus to SARS-CoV-2 [24,25]. However, no intermediate host sample could be obtained in an initial cluster of infection from the Wuhan seafood market, and the earliest symptomatic patients did not have any exposure to the wet market of Wuhan [26]. This left the matter of intermediate host of the virus unresolved, warranting more confirmatory evidence to settle the argument [20].

## 3. Possible mechanism of SARS-CoV-2 invasion into host cells and immune pattern of infection

According to existing clinical data, COVID-19 is not limited to respiratory ailments, but it may also give rise to complications such as acute renal injury and renal necrosis in some patients [27,28]. During the earlier coronavirus outbreak in 2002–2003, SARS-CoV-infected men presented with orchitis as a major complication, which led to reproductive dysfunctions in such men [11]. SARS-CoV and SARS-CoV-2 are similar in that both viruses invade host cells through the ACE2 surface receptor present in the host cell, and it is worth mentioning that ACE2 exists not only in respiratory tissues but also in reproductive tissues, including spermatozoa, seminiferous tubules, Leydig cells and Sertoli cells [29]. This evidence fuelled the possibility of SARS-CoV-2 infection in the male reproductive tract and potential damage to male fertility [30].The expression of ACE2 was also reported in the proximal regions of the heart, kidney, lung, ileum and bladder [31]. Inside the lung, epithelial cells have a higher expression of ACE2. The binding of SARS-CoV-2 with the ACE2 receptor (ACE2-R) allows its entry into cells and completes its replication [6]. This may, in turn, activate direct viral invasion and cause tubular epithelial and podocyte damage, resulting in acute cardiac and lung injury. This is because of the potential SARS-CoV-2-mediated downregulation of ACE2 expression, which may further contribute to an increase in angiotensin 2 (Ang-II)-induced lung injury [6].

In coronaviruses, the entry process is mediated by surface-located spike (S) glycoproteins, which are embedded in the viral envelope [32]. The S protein of SARS-CoV-2 resembles the typical characteristics of the coronavirus S protein, which is divided into two subunits, S1 and S2, responsible for receptor recognition and membrane fusion, respectively. The S1 subunit can be further subdivided into an N-terminal domain (NTD) and a C-terminal domain (CTD). Immunostaining and flow cytometry assays identified the S1 CTD as the key region in SARS-CoV-2 that interacts with the ACE2 receptor. SARS-CoV also uses the S1 CTD as the receptor-binding ligand, and the overall mode of binding is similar to that of the SARS-CoV-2 receptor-binding domain. However, the SARS-CoV-2 CTD has higher atomic interactions with the receptor than the SARS-CoV CTD, which indicates that the SARS-CoV-2 CTD has a higher affinity for the ACE2 receptor [33]. This evidence is important to establish the fact that SARS-CoV-2 is much more infectious than SARS-CoV.

Recent studies have indicated that a particular transmembrane serine protease, designated transmembrane protease/serine subfamily member 2 (TMPRSS2), has a major role in viral entry. ACE2 and TMPRSS2 interact in cellular exocytic pathways and at cell surfaces, resulting in the cleavage of the ACE2 receptor. Proteolysis of the ACE2 receptor by deglycosylation enhances the capability of coronaviruses to enter the cell [34]. SARS-CoV-2 uses the serine protease TMPRSS2 for S protein priming in cells, which significantly increases the cell susceptibility of the virus [35]. ACE2 is highly expressed in spermatogonia, Leydig cells and Sertoli cells, whereas its expression is low in spermatocytes, spermatids and other somatic cells. In a recent study, SARS-CoV-2 RNA was measured in throat and semen samples of infected men. The organ distribution of ACE2 mRNA and protein in human tissue in The Human Protein Atlas Portal revealed relatively high levels of ACE2 protein and RNA expression in the testis [36]. However, the expression of TMPRSS2 is concentrated in spermatogonia and spermatids, with relatively low levels of expression in other cell types. Thus, SARS-CoV-2 may pose a real threat to male fertility due to the expression of both ACE2 and TMPRSS2 in testicular cells [37].

**Figure 2.**
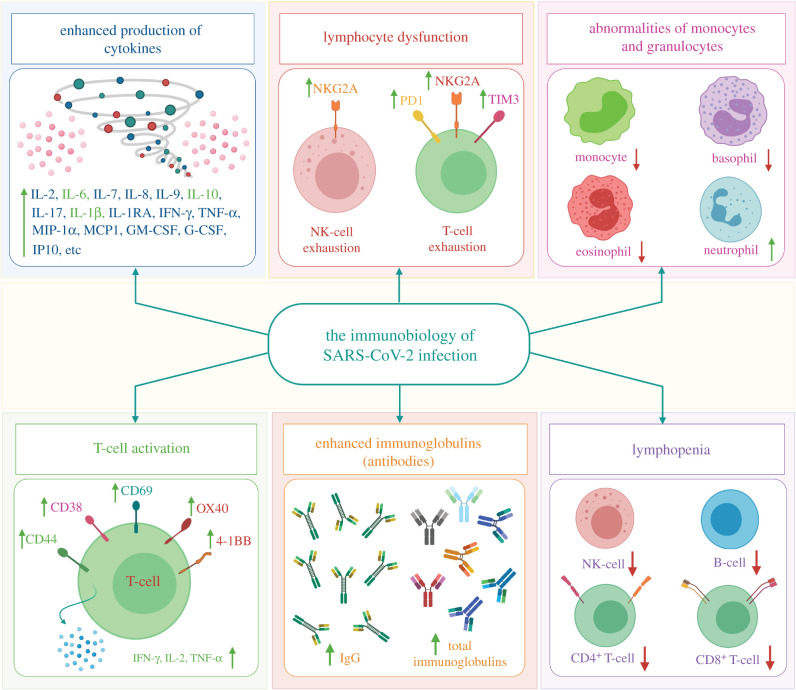
The immunopathology of SARS-CoV-2 infection. SARS-CoV-2 uses the ACE2 receptor to gain entry into the cell (airway epithelial cells), leading to an increase in pro-inflammatory cytokines and the development of cytokine storms, which lead to infection and augment COVID-19 severity. In addition, SARS-CoV-2 infection includes abnormalities of granulocytes and monocytes, lymphopenia, lymphocyte activation and dysfunction, enhanced production of cytokines and increased antibodies [38]. SARS-CoV-2: severe acute respiratory syndrome coronavirus 2, IL: interleukin, TNF-α: tumour necrosis factor alpha, IFN-γ: interferon gamma, MIP-1*α*: macrophage inflammatory protein-1alpha, MCP1: monocyte chemoattractant protein-1, GM-CSF: granulocyte-macrophage colony-stimulating factor, G-CSF: granulocyte colony-stimulating factor, IP10: interferon gamma-induced protein 10, NKG2A: killer cell lectin-like receptor subfamily C member 1, PD1: programmed cell death protein 1, TIM3: T-cell immunoglobulin and mucin domain-3, CD: cluster of differentiation, OX40: secondary costimulatory immune checkpoint molecule, 4–1BB: a member of the tumour necrosis factor receptor superfamily T-cell costimulatory receptor, NK: natural killer.

The immune patterns of SARS-CoV-2 infection include abnormalities of granulocytes and monocytes, lymphopenia, lymphocyte activation and dysfunction, enhanced production of cytokines and increased antibodies (figure 2). Lymphopenia is a key feature of COVID-19 patients, especially in severe cases. CD44, CD69 and CD38 are highly expressed on CD4^+^ and CD8^+^ T cells of patients, and virus-specific T cells from severe cases exhibit a central memory phenotype with high levels of IL-2, TNF-α and IFN-γ. However, lymphocytes show an exhaustion phenotype with killer cell lectin-like receptor subfamily C member 1 (NKG2A), programmed cell death protein-1 (PD1), and T-cell immunoglobulin domain and mucin domain-3 (TIM3) upregulation (figure 2). The percentages of eosinophils, basophils, and monocytes are reduced in severe patients, while neutrophil levels are significantly elevated. Increased cytokine production, especially of IL-6, IL-1β and IL-10, is another key characteristic of SARS-CoV-2 infection and severe COVID-19. IgG levels are also increased, and there is a higher titre of total antibodies [38].

## 4. Effect on the male reproductive system

**Figure 3.**
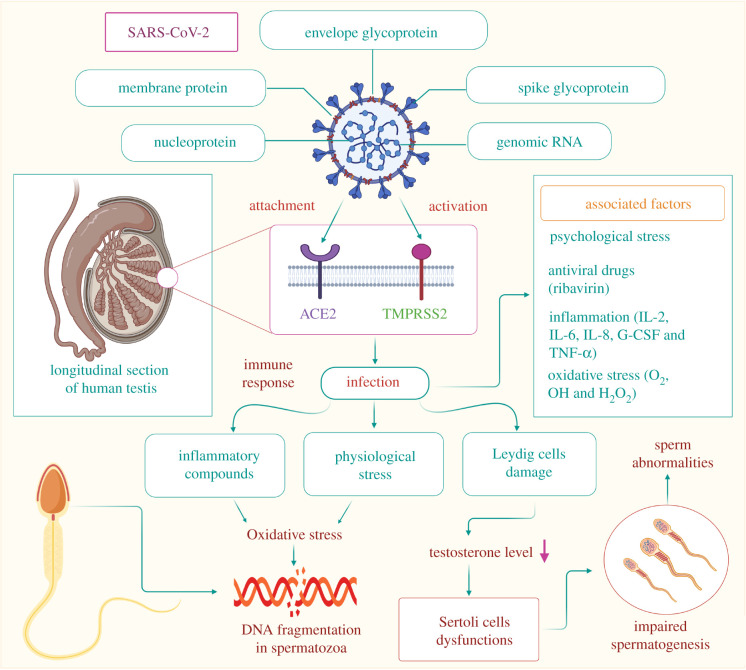
Possible mechanism of SARS-CoV-2 invasion in the reproductive system of infected men and the potential health impacts associated. SARS-CoV-2 gains entry into the reproductive system through the ACE2 and TMPRSS2 receptors present on testicular tissues. The immune response triggered by viral entry produces various inflammatory substances, such as cytokines, which induce OS in testicular cells, which in turn damages the DNA of developing spermatozoa. Various psychological stresses due to SARS-CoV-2 infection may also lead to the production of ROS. SARS-CoV-2 also causes damage to Leydig cells, lowering the production of testosterone, which may ultimately hamper the proper functioning of Sertoli cells. Impaired functioning of Sertoli cells may further disrupt the process of spermatogenesis. However, recent studies have reported low testosterone levels in SARS-CoV-2-infected men with other comorbidities [40,41]. This suggests that normal testosterone may reveal antiviral immune responses to combat SARS-CoV-2 infection in men. SARS-CoV-2: severe acute respiratory syndrome coronavirus 2, ACE2: angiotensin-converting enzyme 2, TMPRSS2: transmembrane protease/serine subfamily member 2, IL: interleukin, G-CSF: granulocyte colony-stimulating factor, TNF-α: tumour necrosis factor alpha, O_2_•: superoxide radical, OH•: hydroxyl radical, H_2_O_2_: hydrogen peroxide.

Several important findings have already been reported regarding the pathology of COVID-19 on the respiratory system as well as other organ systems. Data on the histopathological changes in various other organ systems due to SARS-CoV-2 infection are also emerging [39]. Figure 3 summarizes the potential impact of SARS-CoV-2 infection on the reproductive system in males. As mentioned earlier, the presence of the ACE2 receptor on germ cells, Leydig cells and Sertoli cells recently indicated the testis as a potential target of SARS-CoV-2 infection [29]. The serine protease receptor TMPRSS2 is also present in male reproductive tissues and plays a crucial role in mediating the entry of SARS-CoV-2 [42]. By contrast, another recent study reported a normal testicular appearance in COVID-19 patients [43].

Pathological studies conducted on deceased COVID-19 patients aged 42–87 years confirmed that Sertoli cells are mostly affected, showing swelling, vacuolation, cytoplasmic rarefaction and detachment from the tubular basement membrane. A wide range of changes can be observed in the seminiferous tubules along with a reduction in their number. One major change has been a loss and sloughing of the intratubular cell mass into the lumen. The extent of severity varies from patient to patient, with mild tubular injury in the majority of cases. The number of Leydig cells in the interstitium is also significantly reduced in SARS-CoV-2-infected patients compared to healthy individuals. Various degrees of alterations in spermatogenesis can be observed with considerable consistency in patients of different age groups [5,44]. An immunohistochemical study validated the presence of oedema and mild inflammatory infiltrates composed of CD3-positive T-lymphocytes in the interstitium [44]. It is worth mentioning that viral particles can hardly be traced in the testicular tissues of infected patients. Thus, it may be hypothesized that viral membrane proteins might play a role in injury to seminiferous tubules and Leydig cells. There is also a possibility of hyperthermia due to fever, secondary infection, hypoxia and steroids being the key mediators of testicular damage in SARS-CoV-2 patients [44].

Viral membrane proteins of SARS-CoV-2 may reach the testicular interstitium via blood, and Leydig cells may be one of the first target sites of infection. In the case of extreme viraemia, the virus itself may infiltrate testicular tissues. Invasion by either the virus or its proteins in Leydig cells is followed by alterations in steroidogenic pathways, which may cause Leydig cell dysfunction and a decrease in the ratio of serum testosterone and luteinizing hormone (LH) [45]. SARS-CoV-2-mediated induction of cytokines and chemokines in non-reproductive tissues may be transported to Leydig cells and Sertoli cells. It is likely to be followed by the recruitment of peripheral immune cells, including macrophages and virus-specific T-cells, which may cause inflammation and orchitis [46]. Segmental vascularization of the testis may also account for an orchitis-like syndrome [47]. However, orchitis is not a common symptom reported in COVID-19 patients due to the immunosuppressive properties of Sertoli cells and testicular macrophages, which may play a critical role in suppressing inflammation and limiting virus-associated testicular damage to some extent [48]. Sertoli cells elicit immune privilege to germinal cells with the help of the blood-testis barrier, which includes tight junctions between adjacent Sertoli cells [49]. Apart from working as a barrier between maturing germ cells and immune cells, Sertoli cells can also modulate the immune response by expressing several immunoregulatory factors [50]. However, this immunosuppression is believed to be compromised when there is severe infiltration of viral particles in testicular tissues, as in extreme cases of SARS-CoV-2-associated inflammation. The transient adverse effects on blood-testis barrier integrity may subsequently hamper the normal process of spermatogenesis [48]. This is in contrast to SARS-CoV infection, wherein the elicited immune response is stronger. High fever associated with SARS-CoV infection might add up to the cause of developing orchitis followed by testicular dysfunction. In SARS-CoV-infected testes, leucocyte infiltration has been shown to damage the blood–testis barrier along with the subsequent loss of immune-protective properties [11]. Furthermore, the development of pro-inflammatory cytokines and IgG antibodies in the germinal epithelium, basement membrane, interstitium, vascular endothelium and degenerated germ cells may predispose SARS-CoV-infected men to autoimmune orchitis [11].

Some recent studies have reported the presence of SARS-CoV-2 particles in human semen. Although their route of entry into semen is not fully understood, it may be hypothesized that the virus may reach semen via the impaired blood-testis barrier in the presence of systemic local inflammation. In some patients, viral infiltration into the semen may be manifested by increased viraemia [10]. However, there are contradictory findings, as some other studies failed to detect any viral protein or DNA in the semen of infected COVID-19 patients [8,51]. Hence, it may be suggested that there is a possibility of transmission of the virus from infected men to their female partners during sexual intercourse, although more evidence is necessary to arrive at a definitive conclusion.

Furthermore, SARS-CoV-2 infection has recently been implicated in the disruption of the normal process of autophagy in the testis [30]. Autophagy is believed to aid in the process of degradation of damaged organelles and needless metabolites from the cell apart from elimination of intracellular pathogenic microorganisms, which is of paramount importance. Autophagy is especially important in the reproductive system of men, as it ensures the smooth conduction of spermatogenesis by helping in the formation of specific components and by preventing the unnecessary accumulation of cytoskeleton in sperm cells [52]. Recent studies have found increased expression of autophagy receptor SQSTM1/p62 in SARS-CoV-2-infected cells, thereby suggesting a decrease in autophagic flux [53]. Apart from the virus itself, viral proteins may either induce or inhibit the autophagy pathway directly to achieve viral survival [30]. This suggests that SARS-CoV-2 may limit the level of autophagy, eventually impairing reproductive function in males. The potential health impacts of SARS-CoV-2 infection on the male reproductive system are summarized in table 1.

**Table 1.** Pathophysiology of SARS-CoV-2 infection on the male reproductive system. The specific receptors present in various tissues as well as the immunological response in the form of cytokines elicited by the virus are also highlighted. ACE2, angiotensin-converting enzyme 2; TMPRSS2, transmembrane protease/serine subfamily member 2; IL, interleukin; FGF, fibroblast growth factor; GCSF, granulocyte colony-stimulating factor; GMCSF, granulocyte-macrophage colony-stimulating factor; IFN, interferon; IP, inflammatory protein; MCP, monocyte- chemoattractant protein; MIP, macrophage inflammatory protein; PDGF, platelet-derived growth factor; TNF, tumour necrosis factor; VEGF, vascular endothelial growth factor; ROS, reactive oxygen species; OS, oxidative stress.

	features	references
reproductive tissues showing receptor expression	(i) ACE2: seminiferous tubule, Leydig cells, Sertoli cells, spermatozoa	[42]
	(ii) TMPRSS2: epididymis, prostate gland, seminal vesicles	[52]
immunological response	increase in IL-1β, IL1RA, IL-7, IL-8, IL-9, IL-10, basic FGF, GCSF, GMCSF, IFN-γ, IP10, MCP1, MIP1α, MIP1β, PDGF, TNF-α and VEGF	[54]
effect on reproductive system	*testis*: testicular epithelium damage seminiferous tubules damage Leydig cells and Sertoli cells dysfunction inflammation due to infiltration of pro-inflammatory cytokines hamper in spermatogenesis leading to decreases in sperm count increased ROS production leads to OS, which affects semen parameters, such as sperm function and motility; lipid peroxidation; and DNA damage	[42]

## 5. SARS-CoV-2 and male fertility

These are still early days for understanding the effect of SARS-CoV-2 on male fertility due to the lack of sufficient short- and long-term studies. However, emerging evidence indicates the possibility of testicular damage due to SARS-CoV-2 infection, which in turn may compromise the fertility potential of such men. Both the reproductive and general well-being of patients infected by SARS-CoV-2 may be at risk, as large proportions of vulnerable men are of reproductive age [55]. Proper hormonal balance is an important prerequisite to male fertility potential as well as outcome. Improper endocrine functioning may compromise reproductive health. SARS-CoV-2 is known to induce inflammatory responses that may disrupt the activity of the hypothalamic–pituitary–testicular (HPT) axis, leading to reduced LH, follicle stimulating hormone (FSH) and testosterone levels [42,56]. However, there are contradictions to this theory, as lower serum testosterone levels, higher LH levels and lower testosterone to LH ratio in COVID-19 patients have recently been reported in comparison to healthy men [45]. This exposes the missing links in the association of SARS-CoV-2 infection with modulation of sex hormones, which needs prompt attention to clarify our understanding of SARS-CoV-2 infection and fertility in males.

Oxidative stress (OS) is widely regarded as an important aetiology of male infertility [57–60]. OS is induced when the balance between oxidants and reductants (antioxidants) is disrupted due to increased production of reactive oxygen species (ROS) or reduced generation of the latter. Elevated levels of ROS can affect sperm structural and functional integrity, including motility, morphology, count and viability [61]. High OS is also a threat to sperm DNA integrity, as high ROS concentrations have been linked with DNA fragmentation and chromatin packing. Moreover, the capacity to repair sperm DNA damage is also severely compromised during excessive viraemia, which is attributed to the disruption of the nucleoprotein-mediated defence system that the spermatozoa originally had [62]. This may in turn decrease fertilization rates, reduce implantation, impair embryonic development and increase the rate of pregnancy loss [63,64]. Increased production of ROS is further manifested by the disruption of sperm membrane integration and induction of apoptosis in spermatozoa [65,66]. SARS-CoV-2 can activate oxidant-sensitive pathways via inflammatory responses, thereby inducing OS [42]. As already discussed, this virus has the potential to cause orchitis, which can also trigger disruption of oxidative balance in the testis. According to a study conducted on adult male Sprague–Dawley rats, OS impairs sperm quality even after one complete cycle of spermatogenesis, which is suggestive of its long-term consequences on fertility. It has also been found that the epididymis is largely affected by OS, in contrast to the testis, and it is in the epididymis, where spermatozoa are rendered more vulnerable to oxidative damage [67]. This may be because the developing spermatozoa are somewhat protected in the testes due to the nutritive effects of Sertoli cells and antioxidant protection through superoxide dismutase [68,69]. These observations have been supported by previous studies that concluded that spermatozoa collected from the epididymis of OS-induced rats after 24 h of treatment still had increased DNA oxidation and reduced motility, indicating their long-term effects [70]. From these observations, it may be hypothesized that SARS-CoV-2-induced OS may elicit long-term deleterious effects on male fertility, particularly on developing spermatozoa, but only concrete evidence can settle this argument.

Various side effects conferred by some of the drugs used for the treatment of COVID-19, including ribavirin and glucocorticoids, may serve as a potential aetiology of male infertility [71]. Ribavirin is a broad-spectrum antiviral drug, and as reported in animal experiments, ingestion of this drug resulted in a decrease in testosterone concentration and impairment of spermatogenesis [72]. This drug has also been found to cause sperm abnormalities in rats [73]. As confirmed by clinical studies, ribavirin can cause sperm DNA fragmentation, and when combined with interferon treatment, this antiviral drug can hamper male fertility by decreasing sperm count [74–76]. Glucocorticoids are steroidal drugs that are only used for a short time in COVID-19 patients with progressive deterioration of oxygenation indicators and excessive activation of inflammatory reactions in the body. Small doses of glucocorticoids administered over a short period of time do not have any negative impact on the male reproductive tract, but overdose may expand the interstitial space of the spermatogenic epithelium, followed by destruction of cell connections and the blood-testis barrier, thus making the testicular tissue vulnerable to harmful substances [71].

The rapid global emergence of COVID-19 has created a situation of socio-economic crisis and psychological distress among people across many parts of the world. Modern-day humans are not used to current restriction protocols, and social distancing and isolation regimes often lead to feelings of frustration, stress, anxiety and even depression [77–79]. This forms an important consideration from the perspective of male infertility, as the relationship between stress and infertility has been a topic of serious debate over the years [80]. A prevalence study of psychological symptoms of infertility concluded that 25–60% of infertile individuals report psychiatric symptoms and that their levels of anxiety and depression are significantly higher than those of fertile men [81]. SARS-CoV-2-infected men should be provided with psychological consultation in time to avoid irrational fear and excessive stress, as these may indirectly affect their reproductive health and well-being [77]. Poor fertility potential during psychological stress may be linked with manifestations of lower sperm quality and sexual dysfunctions, which ultimately interfere with the probability of a couple conceiving. Stress and anxiety have been able to influence semen parameters such as lower sperm count and concentration, lower semen volume and higher sperm DNA fragmentation [82,83]. Poor fertility performance in men with psychological disorders is also due to less sexual activity, hypoactive sexual desire and erectile dysfunction [84]. This evidence suggests that SARS-CoV-2-mediated psychological stress may also play an important role in male infertility.

Viral infection might be associated with androgen secretion, and hence an appropriate treatment regimen should consider the androgen levels of the patients [9]. Management strategies such as cryopreservation and assisted reproductive technology (ART) may also be considered vital approaches in tackling specific clinical conditions of male infertility. To employ these strategies for COVID-19 patients, extra precautionary measures should be undertaken during the handling of semen to reduce the chances of viral transmission [85]. Some of the measures for the elimination of the risk of cross-contamination and transmission through cryobanking services include testing both partners for SARS-CoV-2 before initiating treatment, use of closed-carrier cryodevices and sanitary cryostorage protocols [86]. Some embryologists have advocated placing all new cryopreserved specimens into a quarantine tank until patients are determined to have negative viral test results at some future time, especially when dealing with donor semen. Furthermore, all gametes and embryos should go through extensive washing to dilute out potential viral contamination to reduce the possibility of contamination with SARS-CoV-2 [86,87]. The use of high-security straws may also minimize the risks associated with cryopreserving sperm during the pandemic. During the pandemic, a thorough evaluation (especially in the setting of a multidisciplinary team) and molecular confirmation of the absence of SARS-CoV-2 in seminal fluid from asymptomatic cancer patients may assist in ensuring the safety of sperm cryopreservation [88].

## 6. Gender-based susceptibility

Epidemiological studies conducted across different parts of the world have reported higher COVID-19 morbidity and mortality rates in men than in women [89–91]. A recent meta-analysis of 3 111 714 reported global cases also confirmed three times higher demand of intensive treatment unit in male patients as compared to that of female [92]. The vulnerability of men to this disease may be explained by analysing the genetic, immunologic and behavioural differences in both males and females [93].

A positive correlation between ACE2 expression and SARS-CoV-2 infection is already well established. Moreover, there are studies quantifying the expression of ACE2 receptors in human cells based on sex. Single-cell RNA sequencing revealed that males have higher expression of ACE2 in the lungs than females [94]. Another report reported higher circulating levels of ACE2 in healthy and diabetic men as well as in renal disease patients in comparison to women [95]. TMPRSS2 is another protein necessary for SARS-CoV-2 invasion, and its expression has been found to be several-fold higher in prostate epithelium than in other tissues, leaving SARS-CoV-2-infected men more vulnerable to the disease [96].

Immunological studies concluded that sex-based differences contribute to variations in susceptibility to infectious disease and response to vaccines [97]. Experiments conducted in animal models suggested that male mice were more susceptible to SARS-CoV than female mice of similar age, and the enhanced susceptibility was attributed to the accumulation of inflammatory monocytes, macrophages and neutrophils resulting in vascular leakage and alveolar oedema. By contrast, the decreased vulnerability of female mice was probably due to the protective effect of oestrogen receptor signalling [98]. Human studies have also indicated stronger humoural and immune responses against viral infection in females than in males, which holds true in the case of SARS-CoV-2 infection as well [99].

In fact, gender-based differences in behaviour and lifestyle have been considered responsible for the sex-based variation in the pattern of vulnerability to SARS-CoV-2 infection and COVID-19 [84]. Higher smoking and consumption of alcohol among men compared to women may be considered an important factor behind this hypothesis [90]. Recent studies have also reported that women have a more responsible attitude towards the COVID-19 pandemic, which affects their level of compliance with the guidelines issued by the governments and in undertaking preventive measures such as frequent hand washing, using masks and maintaining social distancing protocols, resulting in lower chances of SARS-CoV-2 infection [100].

## 7. Conclusion

Preliminary findings so far suggest the possibility of both direct and indirect infection of SARS-CoV-2 in the reproductive system of males and possible impact on general health and well-being potentially leading to infertility. Evidence indicates a possible long-term effect of the pathogenicity of SARS-CoV-2 infection on testicular tissue, which may further impact reproductive performance. Moreover, the possibility of sexual transmission of SARS-CoV-2 cannot be ruled out.

## 8. Future perspective

The presence of SARS-CoV-2 nuclei has been confirmed in the testicular tissue of infected men using RT-qPCR technique, which is indicative of the direct viral invasion on the male reproductive system [101]. However, the evidence is not yet considered to be conclusive enough to definitely determine as to whether there are asymptomatic patients who need to avoid sexual intercourse with their female partners in order to prevent possible viral transmission [102]. SARS-CoV-2-infected men should be provided with psychological consultation in time to avoid irrational fear and excessive stress, as these may indirectly affect their reproductive health and well-being [71]. The effects of SARS-CoV-2 on the reproductive system of such men may also be elicited by viral infection-mediated immunomodulation and progressive inflammation [103]. Further research is also needed to develop specific treatment strategies for men with an impaired male reproductive system resulting from SARS-CoV-2 infection. In this regard, several therapeutic methods have been developed recently for the treatment of COVID-19 patients, such as mesenchymal stem cells [104], miRNA-based therapy (responsible for changing ACE-2 expression) [105] and hormone therapy [106]. Therefore, treatment regimens should also consider the androgen levels of men, as SARS-CoV-2 infection is believed to be associated with androgen secretion [9]. Management strategies such as cryopreservation and ART may be considered vital approaches in tackling specific clinical conditions of male infertility. To employ these strategies for COVID-19 patients, extra precautionary measures should be undertaken during the handling of semen to reduce the chances of viral transmission [85]. Accordingly, clinical trials should be conducted on SARS-CoV-2-infected male subjects of reproductive age, along with longitudinal studies in paediatric patients to understand the long-term effects of SARS-CoV-2 infection on testicular functions and spermatogenesis in such men [85]. In summary, existing evidence on the impairment of the reproductive system in men who have suffered and/or are suffering from COVID-19 is still preliminary in nature, and further research can only reveal the exact mechanisms and impacts of SARS-CoV-2 infection clearly together with specific short- and long-term approaches for the management of these men.

## Data accessibility

This article has no additional data.
